# Design and Preparation of Antimicrobial Agents

**DOI:** 10.3390/antibiotics11121778

**Published:** 2022-12-08

**Authors:** Martina Hrast

**Affiliations:** Department of Pharmaceutical Chemistry, Faculty of Pharmacy, University of Ljubljana, Aškerčeva cesta 7, 1000 Ljubljana, Slovenia; martina.hrast@ffa.uni-lj.si

Improper use and misuse of antibacterial agents have led to the emergence of (multi)resistant bacterial strains, which are 1 of the top-10 public-health threats, according to the WHO. Antimicrobial resistance was responsible for 1.27 million deaths in 2019. Several common infections, such as gonorrhoea, pneumonia, and tuberculosis, are very difficult or nearly impossible to cure with the current arsenal of antibacterial agents. Therefore, there is an urgent need for the research and development of new antimicrobial agents. There are many different methods for developing new antibacterial agents, i.e., (i) structurally modifying known antibiotics to circumvent the bacterial-resistance mechanism; (ii) developing compounds that inhibit the mechanism of antibiotic resistance; (iii) new antibacterial agents with novel mechanisms of action; and (iv) new compounds that, in combination with (inefficient) antibacterial drugs, restore the effect of these drugs (e.g., efflux pump inhibitors).

This Special Issue contains full research papers and a review article focusing on recent research in the design, preparation, and evaluation of new antibacterial agents with potential uses in human or veterinary medicine.

The aim of the first research published in the Special Issue was to investigate the antimicrobial activity of nineteen 3,6-disubstituted-1,2,4-triazolo[3,4-b]-1,3,4-thiadiazole derivatives [1]. These compounds were tested against a range of Gram-positive and Gram-negative bacteria and six fungi. The minimal inhibitory, bactericidal, and fungicidal concentrations were determined via a microdilution method. All compounds showed antibacterial activity that was more potent than the two reference drugs—ampicillin and streptomycin—against all tested bacteria. The antifungal activity of the compounds was up to 80-fold higher than that of ketoconazole and from 3- to 40-fold higher than that of bifonazole, both of which were used as reference drugs. The most potent compounds were tested for their inhibition of *P. aeruginosa* biofilm formation. One compound showed significantly higher antibiofilm activity and appeared to be equipotent with ampicillin. The prediction of the likely mechanism by docking to antibacterial targets indicated that *E. coli* MurB might be the target, while docking studies to antifungal targets indicated a probable involvement of CYP51 in the mechanism of antifungal activity. Finally, the toxicity tests on human cells confirmed their low toxicity in both the cancer cell line MCF7 and the non-cancer cell line HK-2.

Kolarič et al. [2] described the design and synthesis of a focused library of novel bacterial topoisomerase inhibitors (NBTIs) based on innovative right-hand-side fragments active against DNA gyrase and Topo IV. The novel compounds exhibit very potent and broad-spectrum antibacterial activity, even against some of the most difficult-to-treat pathogens for which new antibacterial agents are urgently needed. They hypothesized that the enzyme activity and whole-cell efficacy of NBTIs depend on the finely tuned relationship between lipophilicity and hydrophilicity that governs the permeability of these compounds across bacterial membranes. The lipophilicity of NBTIs appears to be optimal for passage through the membrane of Gram-positive bacteria, but the higher, although not excessive, lipophilicity and appropriate hydrophilicity appear to be critical for passage through Gram-negative bacterial membranes. However, due to the substitutional hERG inhibition, which is still at least two orders of magnitude away from MICs, further optimization is required to explore their full potential.

Serrano-Aguirre and co-workers [3] focused on the heterologous expression, purification, and characterization of a new *N*-acylhomoserine lactone (AHL) acylase from *Actinoplanes utahensis* NRRL 12,052 (*Au*AHLA) that can hydrolyze a wide range of substrates, including different natural penicillins and several *N*-acyl-L-homoserine lactones, both aliphatic and 3-oxo substituted. Moreover, this enzyme exhibits quorum-quenching activity by inhibiting the production of violacein by *C. violaceum* CV026. Enzymatic synthesis of AHLs represents an alternative method that could be faster, cheaper, and more environmentally friendly than organic synthesis of these compounds. In addition, this enzymatic method could allow for obtaining non-natural analogues of AHLs to block QS receptors and inhibit autoinducer signaling.

The potential synergy between ampicillin and plant antibiotics (pentacyclic triterpenes, ursolic acid (UA) and oleanolic acid (OA)) against MRSA (ATCC33591 and COL) and the mechanisms involved were studied by Verstraeten S. et al. [4]. They monitored the fluorescence of Bodipy-TR-Cadaverin, propidium iodide, and membrane-potential-sensitive probes to determine the ability of UA and OA to bind to lipoteichoic acids (LTAs) and induce membrane permeabilization and depolarization, respectively. Both pentacyclic triterpenes were able to bind to LTA and to induce dose-dependent membrane permeabilization and depolarization. These effects were not accompanied by significant changes in the cellular concentration of pentacyclic triterpenes and/or ampicillin, suggesting a lipid-membrane-mediated effect. They, therefore, focused on membrane effects induced by UA and OA and used membrane models to investigate the role of specific lipids, including phosphatidylglycerol and cardiolipin. Effects on membrane fluidity, permeability, and fusion ability were examined by determining changes in fluorescence anisotropy of DPH/generalized polarization of Laurdan, calcein release from liposomes, fluorescence dequenching of octadecyl-rhodamine B, and liposome size. Both UA and OA showed a dose-dependent effect with membrane rigidification, increase in membrane permeabilization, and fusion. With the exception of the effect on membrane fluidity, the effect of UA was higher than that of OA, suggesting the role of the methyl group position. Overall, the data show that compounds acting on lipid membranes can enhance the effects of other antibiotics, such as ampicillin, and lead to synergistic effects. Such combinations offer the opportunity to explore a larger chemical space for antibiotics.

Rafey and co-workers [5] investigated 26 Ayurvedic medicinal plants traditionally used to treat oral bacterial infections, with the aim of finding new promising antibacterial agents that can be employed for herbal formulation design. Plants were classified into three categories based on their traditional use. All plant extracts were evaluated for their antibacterial, antibiofilm, and antiquorum-sensing properties towards clinical strains isolated from diabetic patients with periodontal disease. The plants with significant activities, including *Juglans regia*, *Syzygium aromaticum*, *Eruca sativa*, *Myristica fragrans*, *Punica granatum*, and *Azadirachta indica*, were further analyzed using HPLC-DAD-QToF and GC-MS. The activity of selected ingredients was evaluated in silico and in vitro. Finally, it could be concluded that eugenol and 2-phenylethylisothiocyanate contribute significantly to the inhibition of bacterial biofilms and quorum sensing.

Frlan evaluated the importance of enzymes belonging to the shikimate pathway as promising targets for antibacterial drugs [6]. In silico analyses combining evolutionary conservation and druggability were performed to determine whether these enzymes are candidates for broad-spectrum antibacterial therapy. The results presented here indicate that the substrate-binding sites of most enzymes in this pathway are suitable drug targets because of their reasonable conservation and druggability scores. An exception was the substrate-binding site of 3-deoxy-*D*-arabino-heptulosonate-7-phosphate synthase, which was found to be undruggable because of its high content of charged residues and extremely high overall polarity. Although the presented study was designed from the perspective of broad-spectrum antibacterial drug development, this workflow can be readily applied to any antimicrobial target analysis, whether narrow or broad spectrum. Moreover, this research also contributes to a deeper understanding of shikimate pathway enzymes and provides valuable insights into their properties.

The design of potential inhibitors to target R67 DHFR was investigated by Silva [7]. First, density-functional calculations were used to identify the modifications of the pterin core that resulted in derivatives that are likely to bind to the enzyme and are not acted upon by the enzyme. These nonreactive molecules were then docked to the active site and the stability of the docking poses of the best candidates was analyzed by triple-molecular-dynamics simulations and compared to the binding stability of the enzyme–substrate complex. The molecule 6-(methoxymethyl)-4-oxo-3,7-dihydro-4*H*-pyrano[2,3-d]pyrimidin-2-yl]methyl-guanidinium was shown to be extremely stable in binding to R67 DHFR by this method and prevented simultaneous binding to the substrate. Additional docking and molecular dynamics simulations indicated that this compound also binds strongly to the canonical prokaryotic dihydrofolate reductase and to human DHFR and, thus, could be a useful tool for the development of chemotherapeutic agents and dual-acting antibiotics targeting the two types of bacterial dihydrofolate reductase.

A library of 2,3-dihydroquinazolin-4(1*H*)-one derivatives was evaluated for in vitro whole-cell antitubercular activity against the tubercular strain H37Rv and multidrug-resistant (MDR) *Mycobacterium tuberculosis* (MTB) strains by Venugopala et al. [8]. Compounds with a di-substituted aryl moiety attached to the 2-position of the scaffold showed a minimum inhibitory concentration (MIC) of 2 µg/mL against the MTB strain H37Rv. Another compound with an imidazole ring at the 2-position of the dihydroquinazolin-4(1*H*)-one core also demonstrated significant inhibitory activity against both the susceptible and MDR strains, with MIC values of 4 and 16 µg/mL, respectively. Computational analysis revealed that the mycobacterial pyridoxal-5’-phosphate (PLP)-dependent aminotransferase (BioA) enzyme could be the potential target for the tested compounds. In vitro ADMET calculations and cytotoxicity studies in the normal human dermal fibroblast cells demonstrated the safety and tolerability of the tested compounds. Thus, these compounds represent a good starting point for further optimization into novel BioA inhibitors for the treatment of drug-sensitive H37Rv and drug-resistant MTB.

Furthermore, Singh et al. [9] compiled a library of 80 compounds from *Ruta graveolens* and citrus plants belonging to the Rutaceae family for pharmacokinetic and molecular docking analyses. The chemical structures of the 80 phytochemicals and the 3D structure of the mycobacterial target InhA enzyme were obtained from the PubChem database and the RCSB Protein Data Bank, respectively. The drug-like properties were evaluated based on Lipinski’s Rule of Five, while the computed phytochemical properties and molecular descriptors were used to predict the ADMET of the compounds. Of these, 11 pharmacokinetically screened compounds were further investigated by performing molecular docking analysis using an InhA as a target. The docking results indicated that gravacridonediol, an important glycosylated natural alkaloid from *Ruta graveolens*, may have promising inhibitory potential against InhA. The binding efficiency of gravacridonediol is supposed to be higher than that of the well-known inhibitor triclosan against the InhA target. The present study shows that the identified natural product gravacridonediol possesses drug-like properties and is also a promising inhibitor of InhA, an important target enzyme of *Mycobacterium tuberculosis*.

Wan et al. [10] designed new inhibitors of the bacterial transferase MraY from *Aquifex aeolicus* (MraY_AA_) based on the central aminoribosyl uridine core of known natural MraY inhibitors. The aim was to obtain an interaction of the oxadiazole linker with the key amino acids (H324 or H325) in the active site of the enzyme, as observed with the highly potent inhibitors carbacaprazamycin, muraymycin D2, and tunicamycin. A group of ten compounds was prepared using a robust microwave-activated one-step sequence for the synthesis of the oxadiazole ring, involving *O*-acylation of an amidoxime and followed by cyclization. The synthetized compounds, with various hydrophobic substituents on the oxadiazole ring, were tested against the MraY_AA_ transferase activity. Although they exhibited low antibacterial activity, nine out of the ten compounds showed inhibition of MraY_AA_ activity in a range of 0.8 µM to 27.5 µM.

In a review paper, Wu et al. [11] focused on the role of *Staphylococcus aureus* YycFG in regulation, biofilm organization, and drug resistance. The YycFG TCS in *S. aureus* is essential for bacterial viability, the regulation of cell membrane metabolism, cell wall synthesis, and biofilm formation. However, the role of YycFG-associated biofilm organization in *S. aureus* antimicrobial resistance and gene regulation has not been discussed in detail. The authors described the main molecules involved in YycFG-associated cell wall biosynthesis, biofilm development, and accumulation of polysaccharide intercellular adhesin (PIA). Two YycFG-associated regulatory mechanisms, accessory gene regulator (*agr*) and staphylococcal accessory regulator (SarA), were also discussed. They underscored the importance of biofilm formation in the development of antibacterial resistance in *S. aureus* infections. The data show that inhibition of the YycFG pathway reduces the production of PIA, biofilm formation, and bacterial pathogenicity, which represents a potential target for the treatment of MRSA-induced infections.

The overview of this Special Issue demonstrates the importance of developing novel inhibitors that target bacterial enzymes. The contributions provide a promising starting point for further development of improved antibacterial agents to inhibit known targets or highlight potential new targets that can be exploited in future antibacterial drug development.
